# Partial Least Squares with Structured Output for Modelling the Metabolomics Data Obtained from Complex Experimental Designs: A Study into the *Y*-Block Coding

**DOI:** 10.3390/metabo6040038

**Published:** 2016-10-28

**Authors:** Yun Xu, Howbeer Muhamadali, Ali Sayqal, Neil Dixon, Royston Goodacre

**Affiliations:** 1 School of Chemistry, Manchester Institute of Biotechnology, The University of Manchester, Manchester M1 7DN, UK; howbeer.muhamadali@manchester.ac.uk (H.M.); Neil.Dixon@manchester.ac.uk (N.D.); roy.goodacre@manchester.ac.uk (R.G.); 2 School of Chemistry, Umm Al-Qura University, Al Taif Road, Mecca 24382, Saudi Arabia; aasayqal@uqu.edu.sa

**Keywords:** partial least squares, structural modelling, experimental design, metabolomics, ***Y*** coding

## Abstract

Partial least squares (PLS) is one of the most commonly used supervised modelling approaches for analysing multivariate metabolomics data. PLS is typically employed as either a regression model (PLS-R) or a classification model (PLS-DA). However, in metabolomics studies it is common to investigate multiple, potentially interacting, factors simultaneously following a specific experimental design. Such data often cannot be considered as a “pure” regression or a classification problem. Nevertheless, these data have often still been treated as a regression or classification problem and this could lead to ambiguous results. In this study, we investigated the feasibility of designing a hybrid target matrix ***Y*** that better reflects the experimental design than simple regression or binary class membership coding commonly used in PLS modelling. The new design of ***Y*** coding was based on the same principle used by structural modelling in machine learning techniques. Two real metabolomics datasets were used as examples to illustrate how the new ***Y*** coding can improve the interpretability of the PLS model compared to classic regression/classification coding.

## 1. Introduction

One of the main research areas in metabolomics is to study the metabolic response to one or a few factors of interest in a given biological system. Design of experiment (DOE) [1] has been widely employed to determine such cause-and-effect relationships. There are many statistical tests to analyse these data generated by different types of DOEs in univariate analyses. However, how to incorporate the DOE information into multivariate modelling is comparatively less well explored. There are several multivariate models proposed in the literature in recent years which can make use of a priori knowledge of DOEs, most notably multilevel simultaneous component analysis (MSCA) [2], analysis of variance (ANOVA)-principal component analysis (ANOVA-PCA) [3], and ANOVA-simultaneous component analysis (ASCA) [4]. These methods share a common methodology, which is to decompose the independent data matrix ***X*** (i.e., the observed data generated by the instruments) to a series of sub-matrices according to the experimental design and perform principal component analysis (PCA) on the decomposed sub-matrices to study the effect of each factor separately. In addition, multi-block models, such as multi-block principal component analysis (MB-PCA) [5], have also been successfully employed to analyse such datasets by repartitioning ***X*** into blocks according to the experimental design, and then performing MB-PCA on the repartitioned multi-block data [6,7]. In addition, multiple supervised models, mostly based on well-known partial least squares (PLS) [1,8] have also been proposed in the literature using a similar methodology, such as priority PLS [9], ANOVA-PLS [10], ANOVA-target projection (ANOVA-TP) [11], and multi-block orthogonal PLS [12]. All of these methods have, to date, focused on processing the ***X*** matrix: where ***X*** is either re-partitioned into blocks according to the experimental design (multi-block approaches) or decomposed into a series of sub-matrices (ANOVA approaches). However, designing the response matrix ***Y*** according to the information in the DOE and to build a supervised model to fit the designed ***Y*** may also be an efficient method to analyse the data generated by the DOE. In fact, this type of method has already been reported previously, albeit in a rather ad hoc manner [13]. In our present study we aim to investigate such a methodology within the framework of structural modelling and propose a workflow for general use.

Historically, the design of output ***Y*** is usually categorised into two types: regression and classification. If the coded output is a series of continuous numbers (e.g., different concentrations of a specific metabolite, time points, temperatures, and so on), these numbers can be directly used as ***Y*** and the corresponding model is called a regression model (e.g., PLS-R). By contrast, if the target is a number of different groups (classes), such as different types of bacteria or different diseases, then ***Y*** is normally coded as a binary matrix in which one column represents one distinct group while each row is the target vector of a sample. A sample of a specific class has its element in the corresponding column coded as “1” and all other elements coded as “0”. The regression models are most suitable for modelling a series of continuous or at least ordinal (e.g., ranks) numbers, while classifications are most suitable for discriminating a set of categories “in parallel”; i.e., there is no particular spatial relationship between these categories.

However, there are cases when neither regression nor classification would be able to present the information in a DOE well. For example, if one conducted an experiment in which two different extraction methods (denoted as E1 and E2) were applied to extract metabolites from three different bacterial cells (denoted as B1, B2, and B3); where the objective was to investigate the differences in metabolic profiles of the three different types of cells and also to compare the extraction efficiency of the two extraction methods. To achieve this objective, a two factor full-factorial experimental design would typically be employed and six different combinations of the two factors need to be examined. This could be considered as a multi-class classification problem having six distinct classes, one for a specific combination of E and B. However, strictly speaking, in this particular case the six classes were probably not truly all “in parallel” because there were pairs of classes, which shared a common factor (e.g., E1B1 vs. E1B2). Therefore, it is reasonable to assume that the E1B1 class should be closer to E1B2 than E2B3. Such a spatial relationship, as a part of the prior knowledge of the experimental design, would be ignored by binary coding.

It has been recognised that not all problems can be explained well in real numbers (regression) or discrete coding (classification) schemes and, sometimes, more general, structured outputs are needed to cope with these data, which cannot be formulated as simple regression or binary classification problems [14]. The key difference between classical regression/classification modelling and structured output modelling is that instead of using a simple error function (e.g., absolute difference between the predicted and known output for regression, correct or wrong (0/1) in predicting a class membership in classification) to evaluate how closely a predicted output matched the expected (target) output, structured output modelling evaluates such qualities using a set of errors. This error set enumerates all possible scenarios, which could happen in the prediction and ensure that there is a sensible gradient between these errors which can reflect the structured nature of the output [14]. Using the same example given above, suppose there is a properly-trained model and one tests it with a group of blind test samples, if an E1B1-labelled sample had been predicted as E2B1 and another E1B1 sample had been predicted as E2B2, then the former prediction should have a lower error than the latter; i.e., the sensible error gradient between the three possible outcomes would be: a correct prediction in both E and B < one wrong prediction in either E or B < wrong predictions in both E and B. A sensible error set for this particular example could be defined as {0,1,2} and each prediction would have an error of one of those numbers in the set. The design of a structured output and the corresponding error set is largely problem-based and it has to be performed based on the available a priori knowledge, then one would need a modelling technique to build a model to establish the relationship between the structural coded output and the observed data and minimise the error derived from the designed error set. Many machine learning models have been extended to model structured data, such as structured support vector machines (S-SVM) [15], deep learning neural networks [16], etc. Compared to those machine learning approaches, PLS has a much larger following in the metabolomics community [17] and almost all major statistical software packages provide easy-to-use PLS routines. As structured output coding only involves designing a more sensible targeted output ***Y*** and the (usually human) interpretations on the predicted outputs afterwards, this can be easily adapted by any software package which supports PLS.

In this study, we explored such a possibility of using a structured output, designed according to the DOE, for PLS modelling and compared the results with classic binary coding. Two recently published real metabolomics datasets were employed which used two different DOEs with different complexity and characteristics.

One dataset (denoted as *riboswitch* in this paper), was obtained from an experiment to investigate the metabolic effects of producing enhanced green fluorescent protein (eGFP) as a recombinant protein in *Escherichia coli* (*E. coli*) cells [18]. A two-factor, full-factorial experimental design was employed and the metabolic profiles of five different *E. coli* strains, namely BL21(DE3) (wild-type), BL21(DE3) pET-empty (PET), BL21(DE3) pET-eGFP (EGFP), BL21(IL3) pET-empty (iL3PET), BL21(IL3) pETeGFP (iL3EGFP), under four different inducer conditions (control, *lac* inducer Isopropyl β-d-1-thiogalactopyranoside (IPTG), pyimido[4,5-*d*] pyrimidine-2,4-diamine (PPDA), and IPTG + PPDA) were measured by Fourier transform infrared (FT-IR) spectroscopy and gas chromatography-mass spectrometry (GC-MS) on cell extracts (the details of this experiment and a more detailed description of the strains can be found in [19]). Since the FT-IR and GC-MS data showed very similar results, only the GC-MS results were reported in this paper. The data will be uploaded and available at MetaboLights.

The other dataset, denoted as *propranolol*, was the results of an experiment investigating the role of efflux pumps in stress tolerance *Pseudomonas putida* exposed to toxic hydrocarbons. In this experiment three different strains of *P. putida* (denoted as DOT-T1E, DOT-T1E-PS28, and DOT-T1E-18) were exposed to four different dosages of propranolol (control, 0.2, 0.4, and 0.6 mg/mL), and three time points were monitored over a period of one hour (start (0), 10, and 60 min after exposure to the drug). At each time point, a sample of cells were collected, extracted, and analysed by GC-MS. The details can be found in [20] and these data are available at MetaboLights under study identifier MTBLS320 [21]. In our previous publications, we employed a series of different MB-PCA models to examine the effect of each factor separately while, in this study, we designed structured outputs to capture the essence of the experimental designs, and used PLS to model these structured outputs so that the effect of the factors of interest can be analysed simultaneously using a single model.

## 2. Results

Since the results from the PLS modelling were in agreement with our previous reports, the detailed biological interpretations and significance of the results of the two datasets can be found in our previous publications (*riboswitch* [19] and *propanolol* [20]) and will not be repeated again in this paper.

### 2.1. Riboswitch

**Figure 1:**
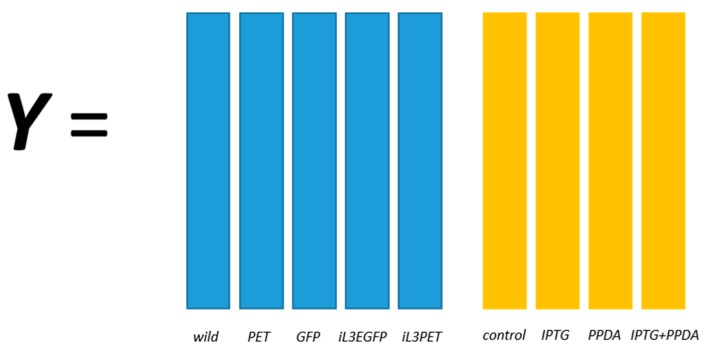
Structured output coding for the *riboswitch* set; for example, a wild-type sample under the IPTG inducer condition would be coded as [1 0 0 0 0 0 1 0 0].

The structured output of this data set is illustrated in Figure 1 and a detailed description is given in Section 4.3.1. The PLS models were validated using a double cross-validation procedure and the reported results were the average of 1000 random splits of training and test sets as described in Section 4.3.2.

The confusion matrix of strain predictions are given in Table 1, while that of inducer condition predictions are shown in Table 2. The overall correct classification rate (CCR) for strain prediction was 80.21%, while that of the inducer condition was 58.20%, suggesting that the strain difference was a more dominating factor compared to the inducer conditions. A more detailed pattern can be revealed by inspecting the two confusion matrices. In the confusion matrix of strain prediction, wild-type and EGFP strains had obtained very high predictive accuracy (approximately 97% and 89%, and also very low off-diagonal misclassification errors of 0.08% and 0%, respectively), indicating that these two strains were most different to each other, while there was minor overlapping between iL3EGFP and iL3PET, which resulted in 8%–19% misclassification errors. This is consistent with the pattern revealed by MB-PCA reported previously [19]. Additionally, in the confusion matrix of inducer condition prediction, there were seemingly two groups. One group consisted of No inducer and PPDA, there was moderate overlapping between these two conditions, and this resulted in 16%–19% misclassification error. IPTG and IPTG + PPDA formed another group and these two conditions overlapped heavily with each other, with misclassification errors as high as 34%. However, the errors between these two groups of conditions were generally lower (no more than 10.3%). This suggested that PPDA had a weak effect on the metabolic profiles of the bacteria cells. IPTG, however, had a very significant impact on the metabolic profile so that IPTG and IPTG + PPDA samples were better separated from those of the other two conditions. Again, this is consistent with the previously reported MB-PCA pattern. We also examined the predictive accuracy of inducer condition prediction on each strain separately, and the CCRs were 45.00%, 40.88%, 66.90%, 50.25%, and 59.58% for wild-type, PET, EGFP, iL3EGFP, and iL3PET, respectively. The much higher CCR in EGFP strains suggested that this strain was more sensitive to the addition of inducers than other strains. The CCRs of strain prediction under each inducer condition were 70.10%, 95.70%, 83.46%, and 72.62%, for control, IPTG, IPTG + PPDA, and PPDA, respectively. Interestingly, PPDA and control had similar CCR, while IPTG had the highest CCR. This also indicated that IPTG had the strongest effect on the metabolic profiles of the bacterial cells and different bacterial strains also responded differently to this inducer. These two sets of CCRs had revealed the delicate interactions between the bacterial strains and inducer conditions. We also employed PLS-DA models using classic binary coding and trained a 20-class classification PLS-DA model. The rearranged confusion matrices (in order to make them comparable with Table 1 and Table 2) are presented in Table 3 and Table 4. It can be seen that similar conclusions can be drawn from those confusion matrices. However, it is interesting to compare the resultant predictive accuracies of these two types of coding. The binary coding resulted in a similar predictive accuracy in predicting inducer conditions (59.37% vs. 58.20%) while that of strain prediction was much lower than the results of the PLS model using structured output (67.78% vs. 80.21%). This may suggest that by using binary coding, the model had focused on separating classes of the “weak” factor (different inducer conditions in this study) and somehow compromised the performance in separating classes of the “strong” factor (strain differences in this study). For binary coding the ***Y*** vectors in PLS-DA, it is also possible to train two separate PLS-DA models, one for each factor. Thus, we have also trained two PLS-DA models, one for strain classification and another for inducer classification. The PLS-DA model focused for strain classification and the average CCR was 78.51%, which is a significant improvement compared to a full 20-class model, but still slightly worse than the model using structured output PLS. For the inducer condition model the averaged CCR was 54.9%, which was the worst prediction accuracy for the three types of coding methods investigated.

### 2.2. Propranolol

**Figure 2:**
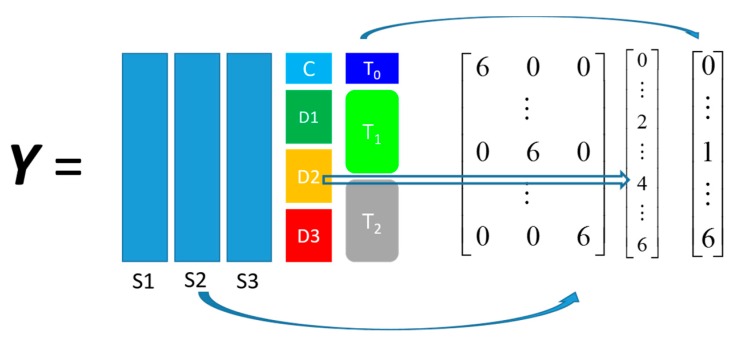
Structured output coding for the *propranolol* set, For example, a sample of S1, D2, and T2 would be coded as [6 0 0 4 6].

For reasons of brevity we denote the three *P. putida* strains: DOT-T1E, DOT-T1E-PS28, and DOT-T1E-18 as S1, S2, and S3, respectively; the four propranolol dosages: control, 0.2, 0.4, and 0.6 mg/mL are denoted as D0, D1, D2, and D3; the three monitored time points: 0, 10, and 60 min after exposure are denoted as T0, T1, and T2. The structured output coding employed for this study is illustrated in Figure 2 and a detailed description is given in Section 4.3.1. The PLS models were also validated by using the same double cross-validation procedure used in the *riboswitch* dataset analysis.

The confusion matrices of strain predictions, dosages, and time point predictions are given in Table 5, Table 6 and Table 7. The confusion matrix of strain classification suggested that S3 (DOT-T1E-18) was most different to the other two strains. The predictive accuracy in dosages prediction showed a gradient from control to D3, suggesting that the effect of the drug increased gradually as the concentrations increased. It appeared that there might be more overlap between D2 and D3, although this was rather inconclusive, probably due to the limited number of samples available. Lastly, the predictive accuracy in the time points suggested that T0 and T1 were more overlapped with each other, and this suggested that the effect of the drug may not be very significant at 10 min after exposure, and it became more pronounced at 60 min after exposure. To examine the further effect of the way coding on the final pattern is revealed by the model we also performed another analysis using an evenly spaced time point coding in which T0, T1, and T2 were coded as 0, 3, and 6 (other coding remained the same). The resultant confusion matrix for time prediction is listed in Table 8. It can be seen that a similar pattern still persisted, except that more T0 samples had been assigned to T1. The unbalanced error between T0 and T1 can be explained by the fact that there were much fewer T0 samples than T1 (T0 only had control samples).

We also performed a PLS-DA using binary coding and treated each unique combination of strain, dosage, and time as a distinct class. The results did not show any sensible pattern and the predictive accuracies were no better than a pure random classifier (data not shown). This could be caused by a large number of classes, limited number of samples, and a mixture nature of ordinal and categorical data in the experimental design. This, again, highlighted the advantage of using a structured output to analyse data from complex experimental designs.

### 2.3. Significant Metabolites Discovery

Identifying significant metabolites is also an important aspect in metabolomics studies and PLS models can provide many statistics which can aid in the discovery of important (input) variables which have contributed to the separation between classes. In this study we employed variable importance in projection (VIP) scores [22] for significant metabolites identification. 

**Figure 3:**
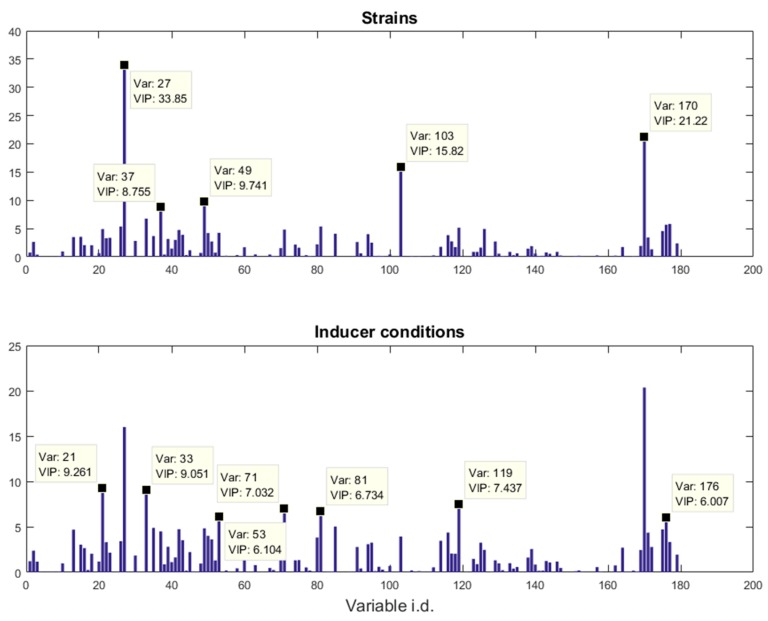
VIP score plots for the *riboswitch* data. The variable identifications and their corresponding VIP scores values were annotated in the data tips. Note that each metabolite is only annotated once, if a metabolite is significant in both VIP score plots (e.g., variable 27), only the higher one is annotated.

The VIP score plots of the *riboswitch* dataset are provided in Figure 3. The top 10 most significant metabolites that could be definitive identified according to MSI [23] (variable 21 and 53, although significant, could not be confidently identified through mass spectra matching) across two blocks in the plot were identified as: glycerol (variable 27), leucine (33), l-isoleucine (37), threonine (49), l-aspartic acid (71), glutamine (81), phosphoric acid (103), lysine (119), silanamine (170), and inosine (176). The box-whisker plots that are presented in Supplementary Materials Figures S1–S10 can be used for visualising the patterns of these metabolites. It is easy to see that most of these metabolites are amino acids and this suggests that the amino acid metabolism had been significantly affected by exposure to different inducers and, also, different strains responded to these inducers differently because these amino acids had significant VIP scores in both blocks. This finding is consistent with our previous report [19]. Among them it is perhaps not surprising that the levels of amino acids, such as threonine, are the highest under inducing conditions (IPTG and IPTG + PPDA) in the protein-producing strain (GFP), while being at its lowest in the wild-type strain, as the protein producers demand higher levels of such amino acids as they require them for synthesis of the recombinant protein. However, the silanamine levels displayed the complete opposite trend, as it is at its highest level in the wild-type while being at its lowest level under the inducing conditions in the protein-producer strains. These findings suggested that, as the wild-type strain is not restricted by GFP production, it may catabolise the available amino acids, as carbon and/or nitrogen sources, to support cellular growth, which results in lower amino acid and higher ammonia levels. By contrast, as the amino acid pools in the protein-producer strains are constricted by the demand for recombinant GFP production, under inducing conditions (IPTG, IPTG + PPDA) these strains may direct the central metabolism towards the required amino acid biosynthetic pathways to keep the homeostasis of these metabolites, which may also result in lower ammonia production and, subsequently, lower silanamine levels.

**Figure 4:**
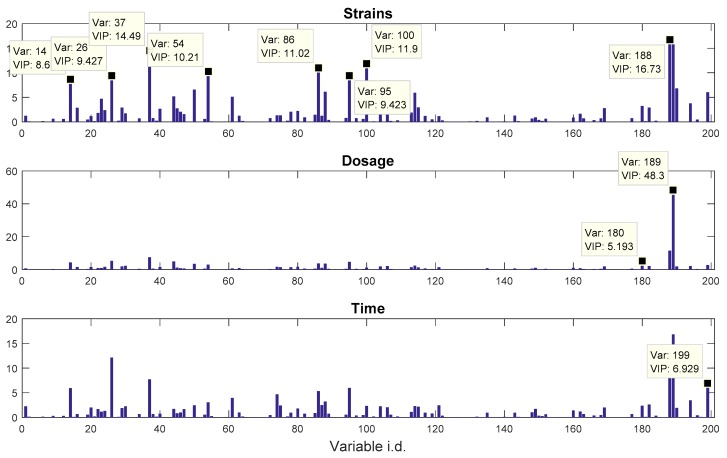
VIP score plots for the propranolol data. The variable identification and their corresponding VIP score values were annotated in the data tips.

The VIP scores plots from PLS-DA analysis on the propranolol dataset are shown in Figure 4. The top 10 most significant and identified metabolites are: alanine (14), butanoic acid (26), leucine (37), serine (54), silanamine (86), phenylalanine (95), ornithine (100), trehalose (188), metoprolol (189), and 5′-adenylic acid (199). The box-whisker plots of alanine, leucine, serine, pheylalanine, and ornithine had already been published in [20] and, therefore, we do not repeat these here. The box-whisker plots of the remaining metabolites are provided in Supplementary Materials Figures S11–S15. It was rather surprising to see that metoprolol had obtained a very significant VIP score and, in fact, is the most significant small molecule compared to all other metabolites. Metoprolol is not a substance of natural occurrence in bacteria, but does have a similar chemical structure to propranolol; we, therefore, suspected that it was converted from the propranolol that had been used to challenge these *P. putida*. We conducted a GC-MS analysis on the propranolol standard and found out that a metoprolol peak was indeed detected within the standard and had a higher peak area than propranolol itself (Supplementary Materials Figure S16). We then conducted LC-MS on the same standard and could only observe a propranolol peak. Thus, we concluded that the majority of propranolol had undergone chemical conversion to metoprolol either during the derivatization process or in the electro-ionisation source used in the GC-MS analysis. This explained the fact that the VIP score of metoprolol had been the highest in the dosage block. It is also worth noting that the propranolol peak (180) had also obtained a significant VIP score of 5.193, even though it is not one of the top 10 highest scores. Additionally, the box-whisker plot of propranolol showed almost exactly the same pattern as that of metoprolol, but at a lower scale (data not shown). The energy-related metabolites, such as trehalose and 5′-adenylic acid (AMP) were also found to be significant. This could be caused by the energy needed to drive the efflux pumps in these *P. putida* strains in response to the exposure of the drug. Such a pattern had also been observed in one of our previous studies [24].

## 3. Discussion

In this paper we have demonstrated that PLS can also be used to model structured outputs and provide improved results over classical binary output coding for modelling data from complex DOEs. It is also easy to implement this methodology, as one only needs to design a structured output ***Y*** based on the experimental design. After modelling these can then be interpreted by producing a series of confusion matrices to gain insights into the modelled patterns in the data. While it is also possible to inspect PLS scores to visualise the pattern, this is usually not easy for PLS using a structured output as the high complexity in ***Y*** usually requires a large number of latent variables (PLS component) to model such complexity sufficiently. Thus, it is not realistic to expect that the overall pattern can always be well represented by, first, a few PLS components, and one may be tempted to plot any latent variables against each other to get the “desired” picture (a practice that is not very objective). Another concern in visualising PLS scores is that it can only present the results of one specific split of the training and test sets while, for robust modelling, it is better to test multiple combinations of training and test sets to get a robust estimation of the errors and prevent getting over optimistic results because of a “lucky” split. Finally, on this point the need to interpret the ***Y*** predictions rather than the PLS scores has been highlighted in [17,25].

The results from the *riboswitch* data suggested that when there is a mixture of strong and weak factors, using binary output coding may result in a model which is focused on separating the classes of the weak factor and compromise its capability in predicting strong factors. Using a structured output coding has resulted in a more balanced model which might still not be able to improve the results of weak factors, although it can significantly improve the results of the strong factors. The results of the *propranolol* dataset have shown that when there is a mixture of values both of an ordinal and categorical nature in the experimental design, structured output coding might be required to be able to successfully build a sensible model. Although it is worth mentioning that the currently proposed method does not explicitly code the interactions between factors. Such effects are inferred from examining the conditional confusion matrices. How to code in interaction terms to ***Y*** and interpret the results is an interesting open research question. Finally, we have demonstrated that the variable importance statistics in PLS modelling, such as VIP scores, can also be used for significant metabolite identification. This can be considered as a major advantage of PLS compared to more “black-boxed” machine learning techniques, such as S-SVM or neural networks.

It is also important to note that compared to those methods which have dedicated structural data modelling, such as S-SVM, PLS has a major limitation in that the model has no flexibility in choosing how to represent the errors, i.e., the difference between coded and predicted outputs. The solution found by PLS models is to maximise the covariance between coded (known target) ***Y*** and the observed ***X*** while methods like S-SVM allows the user-defined error set to be used directly and optimise the model towards minimising such errors. This means that, for PLS, the scale of the coded output will have an influence on the final solution and the results will favour minimising the error of the columns in ***Y*** having larger variance. Therefore, if the structured output has multiple blocks, it is important to ensure that different blocks have comparable variance to prevent the block with the largest variation from dominating the results.

## 4. Materials and Methods

Since these two datasets had already been published elsewhere, only brief descriptions are provided here, and more detailed information about the motivation of the experimental design, bacterial characteristics, sample analysis, and biological interpretations can be found in [19,20].

### 4.1. Riboswitch Experiment

#### 4.1.1. Materials, Strains, and Culture Conditions

Luria Bertani (LB) broth was made from a preparatory mixture (tryptone 10 g/L, NaCl 10 g/L, yeast extract 5 g/L) following the manufacturer’s recommended protocol. The prepared solution was autoclaved and stored at 4 °C.

Five *E. coli* strains were used in this study, coded as wild, PET, EGFP, iL3PET, and iL3EGFP, a detailed description of these strains can be found in [18]. All strains were streak plated three times on LB agar prior to every experiment to ensure the purity of the stocks. One-hundred milligrams per litre of ampicillin and/or 10 mg/L kanamycin were added to the LB broth/agar as selectable plasmid markers where necessary. Starting inocula were prepared by inoculating 25 mL of LB broth with a single colony of the appropriate strain followed by overnight incubation at 37 °C with 200 rpm shaking in a Multitron standard shaker incubator (INFORS-HT Bottmingen Switzerland). Different inducing conditions examined in this study are described in Table 9.

#### 4.1.2. GC-MS Analysis

Fifty millilitres of LB broth, three biological replicates per condition, was inoculated with the appropriate strains using the overnight grown cells to a final OD_600nm_ = 0.1, followed by incubation at 37 °C at 200 rpm shaking for 3 h. Upon reaching the OD_600nm_ = 0.5 the samples were exposed to one of the inducing conditions (Table 9), and the incubation temperature was decreased to 20 °C at 200 rpm for 8 h in shaking incubators, which sums up to a total of 11 h of incubation. Fifteen millilitre samples from each flask were quenched using 30 mL, 60% aqueous methanol (−48 °C) following procedures described in previous studies [26]. The extraction protocol was also adapted from [26] with the exception of centrifugation speed being set at 15,871× *g*. All extracts were normalized according to OD_600nm_ followed by combining 100 µL from each of the samples in a new tube, to be used as the quality control (QC) sample. One-hundred microlitre internal standard solution (0.2 mg/mL succinic-*d*_4_ acid, 0.2 mg/mL glycine-*d*_5_, 0.2 mg/mL benzoic-*d*_5_ acid, and 0.2 mg/mL lysine-*d*_4_) was added to all the samples (including QCs) followed by an overnight drying step using a speed vacuum concentrator (Concentrator 5301, Eppendorf, Cambridge, UK).

Derivatization was carried out by oximation (using methoxyamine-hydrochloride in pyridine) followed by a silylation step (using *N*-methyl-*N*-(trimethylsilyl) trifluoroacetamide), as described by [27,28].

The derivatized samples were randomised and analysed using a Gerstel MPS-2 autosampler (Gerstel, Baltimore, MD, USA) used in conjunction with an Agilent 6890N GC oven (Wokingham, UK) coupled to a Leco Pegasus III mass spectrometer (St. Joseph, MI, USA) following previously published methods [29,30]. Collected data were deconvolved using Leco ChromaTOF software v3.32 and initial identification was carried out according to metabolomics standards initiative (MSI) guidelines [23] followed by removal of mass spectral features with high deviation within the QC samples [29]. The chromatographic peaks corresponding to PPDA and IPTG were also removed from the data before subjecting the data to PLS to eliminate any variation that might result from the presence of these compounds.

### 4.2. Propranolol Experiment

#### 4.2.1. Materials, Strains, and Culture Conditions

Three bacterial strains of *P. putida* DOT-T1E were used in this study, denoted as DOT-T1E, DOT-T1E-PS28, and DOT-T1E-18; their relevant characteristics, and references for further information on each strain can be found in [31,32,33]. All strains were sub-cultured in triplicate to obtain axenic cultures. Individual colonies were then picked and transferred from plates into 250 mL flasks containing 50 mL of autoclaved Lysogeny broth (LyB) medium and incubated at 24 h at 30 °C in an orbital incubator (Infors HT Ltd., Reigate, UK) shaken at 200 rpm.

#### 4.2.2. Sample Collection and GC-MS Analysis

Cells were grown in 50 mL of LyB medium for 5 h at 30 °C and 200 rpm. Once cell cultures reached the mid-exponential phase, samples were divided into two groups. One group was kept as a control and to the second group propranolol was added at three different concentrations (0.2, 0.4, and 0.6 mg/mL). These cultures were then incubated for an additional 8 h. Fifteen millilitre samples were quenched at three time points 0, 10, and 60 min before and after the addition of propranolol (0 min refers to the point immediately before the addition of propranolol). This procedure was performed with four biological replicates.

The samples (15 mL) were plunged into a double volume of 60% cold methanol (−50 °C) in a 50 mL tube. The quenched culture mixture was centrifuged (3000× *g*, 10 min, 1 °C), and then the supernatant was discarded, while the cell pellets were stored at −80 °C until required for metabolite extraction.

The biomass pellets were resuspended in 750 μL of freshly prepared cold methanol (80%). The solution was then transferred to a 2 mL Eppendorf microcentrifuge tube. This was followed by a freeze-thaw cycle in order to extract the intracellular polar metabolites from the cells. Samples were centrifuged at (13,500× *g*, 3 min, 4 °C) and the supernatant was transferred to new tubes and stored on dry ice. The extraction was performed again on the remaining pellet and both supernatants were combined and again stored on dry ice. A final aliquot (1400 μL) of metabolite extracts were normalised using 80% methanol according to OD at 660 nm. A quality control (QC) sample was prepared by transferring 100 μL from each of the samples to a new (15 mL) centrifuge tube. This was followed by the addition of (100 μL) of internal standard solution (0.2 mg/mL glycine-*d*_5_, 0.2 mg/mL benzoic-*d*_5_ acid, 0.2 mg/mL lysine-*d*_4_, and 0.2 mg/mL succinic-*d*_4_ acid) to all samples. The samples were lyophilized for 16 h by speed vacuum concentrator (concentrator 5301; Eppendorf, Cambridge, UK), and then the pellet was stored at −80 °C for further analysis.

Prior to GC-MS analysis the samples were derivatized using the same method used in *riboswitch* experiment. These samples were then randomised and analysed by using gas chromatography electron ionisation time-of-flight mass spectrometry (GC-TOF-MS) using an Agilent 6890 GC instrument coupled to a LECO Pegasus III TOF mass spectrometer (Leco, St. Joseph, MI, USA), as described previously [26,27,28]. A GC column (VF-17MS column, 0.25 mm ID × 30 m × 0.25 μm film thickness, Varian, cat. No. CP8982) was employed at a constant helium carrier gas flow of 1 mL/min, with a temperature program starts at 70 °C and end at 300 °C. The mass spectrometer source was operated at a temperature of 250 °C in electron ionization (EI) mode, with an electron energy of 70 eV and the detector is operated in the range 1400–1800 V. Raw data processing was undertaken using LECO ChromaTOF v3.26 in order to construct a data matrix of metabolite peak vs. sample and infilled with peak areas for metabolites that were detected. A reference database was prepared that contained retention times, quant mass, peak area, retention index value, and peak number related to each peak by analysing QC samples. The identification of analytes was based on both spectral similarity and matched with retention indices. An in-house library, as well as the NIST library, was used for identification, and we also followed the same MSI guidelines for metabolite identification as for the *riboswitch* experiment.

### 4.3. PLS Modelling

All of the data analysis were performed using in-house scripts written in MATLAB 2014a (Mathworks, MA, USA) environment and these scripts are freely available online at [34]. Both datasets were firstly aligned using QCs [29], and the missing values were imputed by using KNN-imputation method [35] and then subjected to PLS analysis.

#### 4.3.1. Structured Output Coding, Error Evaluation and Results Interpretation

##### • *Riboswitch* Data

The structured coding for this dataset is relatively straightforward. It can be considered as a classification model that will be calibrated to predict two class membership *simultaneously*. Therefore, the structure coded output is a combination of two binary matrices, one for bacterial strains and another for inducer conditions, as illustrated in Figure 1. The predicted output was firstly “crisped” so that an unambiguous class membership for each class (strain and inducer condition) could be more readily assigned. This is done by assigning the class membership to the corresponding column which achieved the highest number compare to other columns within the block. The error set was a record of the count of the number of blocks which had been misclassified which varied from 0 (correct in both blocks), 1 (wrong in either strain or inducer condition block), to 2 (wrong in both blocks).

##### • *Propranolol* Data

The structured coding for *propranolol* dataset was more complicated as it consisted of three blocks: one classification block (strains) and two ordinal blocks (dosage and time). These three blocks were also at different scales in which the strain block could be presented by a dummy binary matrix in any scale, the dosage varied from 0–0.6 mg/L while the time block varied from 0–60 min. These three blocks need to be properly balanced to avoid one block with the largest variance dominating other “minor” blocks because PLS can only minimise the differences between the predicted and known outputs in least square sense. In this study we employed the following coding scheme: (1) the two discrete numbers in the binary matrix part were coded as “0” and “6”; (2) the four different dosages were coded 0, 0.2, 0.4, and 0.6 mg/L as 0, 2, 4, and 6, respectively; and (3) the three time points were coded as 0, 1, and 6 respectively as illustrated in Figure 2.

This coding method ensured that the “worst” predictions in different blocks would result in similar errors, e.g., a “complete” misclassification error in strain prediction, D0 had been predicted as D3 or T2 had been predicted as T0. The coded values in the dosage column were evenly spaced as in the experiment the dosages applied were also evenly spaced while the unevenly spaced intervals in the time column had reflected the real-time differences, but at a different scale, which is comparable with other blocks.

The interpretation of the predicted outputs was also performed on three blocks separately, and the classification block was interpreted in the same way as what had been done for the *riboswitch* dataset. The interpretations on the two ordinal blocks also followed a similar “crisping” of the output. For each sample, the predicted value was compared to all available known values, i.e., 0, 2, 4, and 6, for the dosage column, and 0, 1, and 6 for the time column. The one with the smallest absolute difference was then assigned to the corresponding sample. For example, if a sample had a predicted dosage as 2.5 and time as 0.8, this sample would be assigned as D1 and T1. The reason is that it was not easy to infer the spatial distribution of different types of samples using regression-based indicators, such as root mean squared error (RMSE), *R*^2^, or *Q*^2^ [1]. Additionally, the number of different points were very limited (four for dosage and only three for time), a plot of predicted vs. known values could not show a clear monotonic changing trend, either. By assigning the “raw” outputs in prediction to the nearest target, a confusion matrix can be calculated and the types of samples with larger misclassification error between each other can be considered as closer-related types, and vice versa. 

The error set also consists of the possible number of misclassification/misassignment of the blocks, which are 0, 1, 2, and 3.

#### 4.3.2. PLS Modelling

A double cross-validation (CV) procedure [25] was employed to train and validate the PLS models. The split of training and test set was based on biological replicates. For example, in the *riboswitch* experiment, there were three biological replicates for each of the 5 × 4 = 20 different combinations of the two factors (five strains, and four inducer conditions). This resulted in 20 × 3 = 60 samples in total. For one split of the training and test set, one biological replicate from each unique combination of strain and inducer condition was randomly selected and removed from the data to form a blind test set which had 20 samples. The model was then built on the remaining 40 samples and the optimal number of LVs was chosen by performing a LOOCV on the training set. The CV errors were calculated using the corresponding error set. The final PLS model was then built on the whole training set using the optimal number of PLS components, which had minimal cross-validation error, and this model was then applied to the blind test set to generate a predicted output. This output was then compared to the known structured output, as described before, to calculate the misclassification or misassignment errors. A confusion matrix was then calculated in which each row represents the percentage of a type of samples that had been predicted as one of all the available types. This procedure had been repeated 1000 times, where in each iteration a different training and test set had been randomly chosen as described above. The confusion matrices of these 1000 iterations were averaged to generate the final confusion matrix. Additionally, in order to assess the statistical significance of the effect of each factor, we conducted permutation tests [25]. For each split of the training and test sets, the labels of the samples were randomly permuted and the PLS model was built and validated using the same procedure as above. The predictive error in the test set was then calculated and recorded. An empirical *p*-value was derived by calculating the ratio of the number of cases when the errors of the models using the known labels (observed errors) had been higher than the ones using randomly-permuted labels (null errors) over the all 1000 iterations. A low *p*-value (e.g., *p* < 0.01, meaning that there were less than 10 cases out of 1000 in which the observed errors were higher than the corresponding null errors) would suggest the effect of the factor was statistically significant, while the effect would be considered as insignificant if the *p*-value was high (e.g., *p* > 0.05).

Mean centre pre-processing was applied for the PLS modelling [1]. On the training set, the means of both ***X*** and ***Y*** matrices (denoted as $\bar{x}$ and $\bar{y}$, respectively) were calculated, recorded, and subtracted from ***X*** and ***Y***, respectively. The PLS model was then built between the mean centred ***X*** and ***Y***. Then, in the validation or blind test, $\bar{x}$ was subtracted from ***X*** in the validation/test set and the trained PLS model was applied to calculate the predicted ***Y*** (denoted as $\hat{Y}$). The final prediction was $\hat{Y}$ with $\bar{y}$ added back into it.

#### 4.3.3. VIP Scores for Significant Metabolite Identification

For PLS with multiple columns of outputs, there is a VIP score vector for each column in ***Y***. To simplify the task of inspection the VIP scores were summarised according to the blocks. This is done by taking the maximum of the VIP scores of the ***Y*** variables within the group. For the *riboswitch* dataset, the VIP scores for strain classification were the maximum VIP scores of the first five columns, and those for inducer condition classification were the maximum VIP scores of the last four columns. For the *propranolol* dataset, the VIP scores for the strain classification were the maximum VIP scores of the first three columns; those for dosage and time modelling were the VIP scores of the fourth and fifth column, respectively.

## 5. Conclusions

In conclusion, we have demonstrated that it is possible to implement a structured output for modelling metabolomics data when multiple interacting factors are present in the experimental design. We believe that this approach would have general utility in metabolomics data analysis as well as in other areas where the analysis of complex multivariate data is needed.

## Figures and Tables

**Table 1:** PLS predictions of the *riboswitch* set. Confusion matrix of *strain* prediction using *structured output*.

	Wild-Type	PET	EGFP	iL3EGFP	iL3PET
Wild-type	97.20%	0.38%	0.08%	1.73%	0.63%
PET	10.03%	71.93%	8.20%	6.23%	3.63%
EGFP	0.00%	2.88%	89.33%	4.83%	2.98%
iL3EGFP	1.20%	8.53%	2.35%	69.10%	18.83%
iL3PET	3.35%	12.55%	2.85%	7.78%	73.48%

Overall CCR = 80.21% (*p* < 0.001) (CCR = correct classification rate).

**Table 2:** PLS predictions of the *riboswitch* set. Confusion matrix of *inducer* condition prediction using *structured output*.

	No Inducer	IPTG	IPTG + PPDA	PPDA
No inducer	66.62%	6.56%	10.92%	15.90%
IPTG	12.66%	58.04%	22.58%	6.72%
IPTG + PPDA	4.02%	34.42%	44.40%	17.16%
PPDA	19.14%	6.58%	10.26%	64.02%

Overall CCR = 58.20% (*p* < 0.01).

**Table 3:** PLS predictions of the *riboswitch* set. Confusion matrix of *strain* prediction using *binary coding*.

	Wild-Type	PET	EGFP	iL3EGFP	iL3PET
Wild-type	79.53%	4.40%	1.58%	9.55%	4.95%
PET	8.93%	52.88%	11.53%	18.25%	8.43%
EGFP	0.70%	8.55%	83.23%	3.65%	3.88%
iL3EGFP	6.53%	8.28%	5.60%	62.08%	17.53%
iL3PET	7.43%	10.00%	10.25%	6.13%	66.20%

Overall CCR = 67.78% (*p* < 0.001).

**Table 4:** PLS predictions of the *riboswitch* set. Confusion matrix of *inducer condition* prediction using *binary coding*.

	No Inducer	IPTG	IPTG + PPDA	PPDA
No inducer	64.60%	7.10%	12.16%	16.14%
IPTG	10.44%	59.40%	22.50%	7.66%
IPTG + PPDA	7.58%	34.44%	43.46%	14.52%
PPDA	15.38%	5.00%	9.60%	70.02%

Overall CCR = 59.37% (*p* < 0.01).

**Table 5:** PLS predictions for the *propranolol* set. Confusion matrix for *strain* prediction.

	S1	S2	S3
S1	78.03%	21.26%	0.71%
S2	10.40%	87.67%	1.93%
S3	4.78%	3.22%	92.00%

Overall CCR = 85.90% (*p* < 0.001).

**Table 6:** PLS predictions for the *propranolol* set. Confusion matrix for *dosages of propranolol* prediction.

	D0	D1	D2	D3
D0	99.08%	0.91%	0.01%	0%
D1	0.20%	64.45%	35.35%	0%
D2	0%	0.05%	93.23%	6.72%
D3	1.67%	0.10%	39.35%	58.88%

Overall accuracy = 78.91% (*p* < 0.001).

**Table 7:** PLS predictions for the *propranolol* set. Confusion matrix for *time point* prediction.

	T0	T1	T2
T0	30.13%	55.57%	14.30%
T1	15.60%	58.35%	26.05%
T2	1.48%	27.15%	71.37%

Overall accuracy = 52.97% (*p* < 0.01).

**Table 8:** PLS predictions for the *propranolol* set. Confusion matrix for *time point* prediction using an evenly spaced coding.

	T0	T1	T2
T0	20.64%	70.40%	8.96%
T1	7.15%	67.78%	25.07%
T2	0.78%	35.72%	63.50%

Overall accuracy = 49.30% (*p* < 0.01).

**Table 9:** Different inducer conditions.

Inducer Compound	Final Concentration
0.9% NaCl solution (control, no inducer)	-
IPTG (*lac* inducer)	50 μM
PPDA (riboswitch inducer ligand)	200 μM
IPTG + PPDA	50 μM + 200 μM

## Supplementary Materials

The following are available online at http://www.mdpi.com/2218-1989/6/4/38/s1, Figure S1: Box-whisker plot of alanine in *riboswitch* data set; Figure S2: Box-whisker plot of glycerol in *riboswitch* data set; Figure S3: Box-whisker plot of threonine in *riboswitch* data set; Figure S4: Box-whisker plot of glutamine in *riboswitch* data set; Figure S5: Box-whisker plot of pyroglutamic acid in *riboswitch* data set; Figure S6: Box-whisker plot of lysine in *riboswitch* data set; Figure S7: Box-whisker plot of phosphoric acid in *riboswitch* data set; Figure S8: Box-whisker plot of silanamine in *riboswitch* data set; Figure S9: Box-whisker plot of ltryptophan in *riboswitch* data set; Figure S10: Box-whisker plot of leucine in *riboswitch* data set; Figure S11: Box-whisker plot of butanoic acid in *propranolol* data set; Figure S12: Box-whisker plot of silanamine in *propranolol* data set; Figure S13: Box-whisker plot of trehalose in *propranolol* data set; Figure S14: Box-whisker plot of metoprolol in *propranolol* data set; Figure S15: Box-whisker plot of 5′-adenylic acid in *propranolol* data set; Figure S16: GC-MS analysis on propranolol standards.

## References

[1] Brereton R.G. (2003). Chemoemtrics: Data Analysis for the Laboratory and Chemical Plant.

[2] Timmerman M.E. (2006). Multilevel component analysis. Br. J. Math. Stat. Psychol..

[3] Harrington P.B., Vieira N.E., Espinoza J., Nien J.K., Romero R., Yergey A.L. (2005). Analysis of variance-principal component analysis: A soft tool for proteomic discovery. Anal. Chim. Acta.

[4] Smilde A.K., Jansen J.J., Hoefsloot H.C.J., Lamers R.-J.A.N., van der Greef J., Timmerman M.E. (2005). ANOVA-simultaneous component analysis (ASCA): A new tool for analysing designed metabolomics data. Bioinformatics.

[5] Smilde A.K., Westerhuis J.A., de Jong S. (2003). A framework for sequential multiblock component methods. J. Chemometr..

[6] Kassama Y., Xu Y., Dunn W.B., Geukens N., Anné J., Goodacre R. (2010). Assessment of adaptive focused acoustics versus manual vortex/freeze-thaw for intracellular metabolite extraction from Streptomyces lividans producing recombinant proteins using GC-MS and multiblock principal component analysis. Analyst.

[7] Xu Y., Cheung W., Winder C.L., Goodacre R. (2010). VOC-based metabolic profiling for food spoilage detection with the application to detecting Salmonella typhimurimum contaminated pork. Anal. Bioanal. Chem..

[8] Wold S., Sjöström M., Eriksson L. (2001). PLS-regression: A basic tool of chemometrics. Chemometr. Intell. Lab..

[9] Höskuldsson A. (1996). Experimental design and priority PLS regression. J. Chemometr..

[10] Thissen U., Wopereis S., van den Berg S.A., Bobeldijk I., Kleemann R., Kooistra T., van Dijk K.W., van Ommen B., Smilde A.K. (2009). Improving the analysis of designed studies by combining statistical modelling with study design information. BMC Bioinform..

[11] Marini F., de Beer D., Joubert E., Walczak B. (2015). Analysis of variance of designed chromatographic data sets: The analysis of variance-target projection approach. J. Chromatogr. A.

[12] Boccard J., Rudaz S. (2016). Exploring Omics data from designed experiments using analysis of variance multiblock Orthogonal Partial Least Squares. Anal. Chim. Acta.

[13] Martens M., Bredie W.L.P., Martens H. (2000). Sensory profiling data studied by partial least squares regression. Food Qual. Prefer..

[14] Bakir G., Taskar B., Hofmann T., Schölkopf B., Smola A., Vishwanathan S.V.N. (2007). Predicting Structured Data.

[15] Tsochantaridis I., Joachims T., Hofmann T., Altun Y. (2005). Large Margin Methods for Structured and Interdependent Output Variables. J. Mach. Learn. Res..

[16] Schulz H., Behnke S., Wermter S. (2014). Structured Prediction for Object Detection in Deep Neural Networks. Artificial Neural Networks and Machine Learning—iCANN 2014.

[17] Gromski P.S., Muhamadali H., Ellis D.I., Xu Y., Correa E., Turner M.L., Goodacre R. (2015). A tutorial review: Metabolomics and partial least squares-discriminant analysis—A marriage of convenience or a shotgun wedding. Anal. Chim. Acta.

[18] Morra R., Shankar J., Robinson C., Halliwell S., Butler L., Upton M., Hay S., Micklefield J., Dixon N. (2016). Dual transcriptional-translational cascade permits cellular level tuneable expression control. Nucl. Acids Res..

[19] Muhamadali H., Xu Y., Morra R., Trivedi D.K., Rattray N.J.W., Dixon N., Goodacre R. (2016). Metabolomic analysis of riboswitch containing *E. coli* recombinant expression system. Mol. Biosyst..

[20] Sayqal A., Xu Y., Trivedi D.K., AlMasoud N., Ellis D.I., Rattray N.J.W., Goodacre R. (2016). Metabolomics analysis reveals the participation of efflux pumps and ornithine in the response of *Pseudomonas putida* DOT-T1E cells to challenge with propranolol. PLoS ONE.

[21] MTBLS320: Metabolomics Analysis Reveals the Participation of Efflux Pumps and Ornithine in the Response of *Pseudomonas putida* DOT-T1E Cells to Challenge with Propranolol. http://www.ebi.ac.uk/metabolights/MTBLS320.

[22] Chong I., Jun C. (2005). Performance of some variable selection methods when multicollinearity is present. Chemometr. Intell. Lab..

[23] Sumner L.W., Amberg A., Barrett D., Beger R., Beale M.H., Daykin C., Fan T.W.-M., Fiehn O., Goodacre R., Griffin J.L. (2007). Proposed minimum reporting standards for chemical analysis. Metabolomics.

[24] Currie F., Broadhurst D.I., Dunn W.B., Sellick C.A., Goodacre R. (2016). Metabolomics reveals the physiological response of *Pseudomonas putida* KT2440 (UWC1) after pharmaceutical exposure. Mol. Biosyst..

[25] Westerhuis J.A., Hoefsloot H.C.J., Smit S., Vis D.J., Smilde A.K., van Velzen E.J.J., van Duijnhoven J.P.M., van Dorsten F.A. (2008). Assessment of PLSDA cross validation. Metabolomics.

[26] Winder C.L., Dunn W.B., Schuler S., Broadhurst D., Jarvis R., Stephens G.M., Goodacre R. (2008). Global metabolic profiling of *Escherichia coli* cultures: An evaluation of methods for quenching and extraction and intracellular metabolites. Anal. Chem..

[27] Wedge D.C., Allwood J.W., Dunn W., Vaughan A.A., Simpson K., Brown M., Priest L., Blackhall F.H., Whetton A.D., Dive C. (2011). Is serum or plasma more appropriate for intersubject comparisons in metabolomics studies? An assessment in patients with small-cell lung cancer. Anal. Chem..

[28] Fiehn O., Kopka J., Trethewey R.N., Willmitzer L. (2000). Identification of Uncommon Plant Metabolites Based on Calculation of Elemental Compositions Using Gas Chromatography and Quadrupole Mass Spectrometry. Anal. Chem..

[29] Begley P., Francis-McIntyre S., Dunn W.B., Broadhurst D.I., Halsall A., Tseng A., Knowles J., Goodacre R., Kell D.B. (2009). Development and performance of a GC-TOF-MS analysis for large-scale untargeted metabolomic studies of human serum. Anal. Chem..

[30] Dunn W.B., Broadhurst D., Begley P., Zelena E., Francis-McIntyre S., Anderson N., Brown M., Knowles J.D., Halsall A., Haselden J.N. (2011). Procedures for large-scale metabolic profiling of serum and plasma using gas chromatography and liquid chromatography coupled to mass spectrometry. Nat. Protoc..

[31] Ramos J.L., Duque E., Huertas M.J., Haidour A. (1995). Isolation and expansion of the catabolic potential of a Pseudomonas-putida strain able to grow in the presence of high concentrations of aromatic-hydrocarbons. J. Bacteriol..

[32] Ramos J.L., Duque E., Godoy P., Segura A. (1998). Efflux pumps involved in toluene tolerance in *Pseudomonas putida* DOT-T1E. J. Bacteriol..

[33] Rojas A., Duque E., Mosqueda G., Golden G., Hurtado A., Ramos J.L., Segura A. (2001). Three efflux pumps are required to provide efficient tolerance to toluene in *Pseudomonas putida* DOT-T1E. J. Bacteriol..

[34] Biospec/cluster-toolbox-v2.0. https://github.com/Biospec/cluster-toolbox-v2.0.

[35] Troyanskaya O., Cantor M., Sherlock G., Brown P., Hastie T., Tibshirani R., Botstein D., Altman R. (2001). Missing value estimation methods for DNA microarrays. Bioinformatics.

