# Supplementary material for: Partial Least Squares with Structured Output for Modelling the Metabolomics Data Obtained from Complex Experimental Designs: A Study into the Y-Block Coding
Source: Metabolites. 2016 Oct 28;6(4):38. doi: 10.3390/metabo6040038 (PMC5192444; doi:10.3390/metabo6040038)
Supplement: Supplementary file 1 [file metabolites-06-00038-s001.pdf]

Supplementary Materials: Partial Least Squares with Structured Output for Modelling the Metabolomics Data Obtained from Complex Experimental Designs: A Study into the *Y*-Block Coding

Yun Xu, Howbeer Muhamadali, Ali Sayqal, Neil Dixon and Royston Goodacre

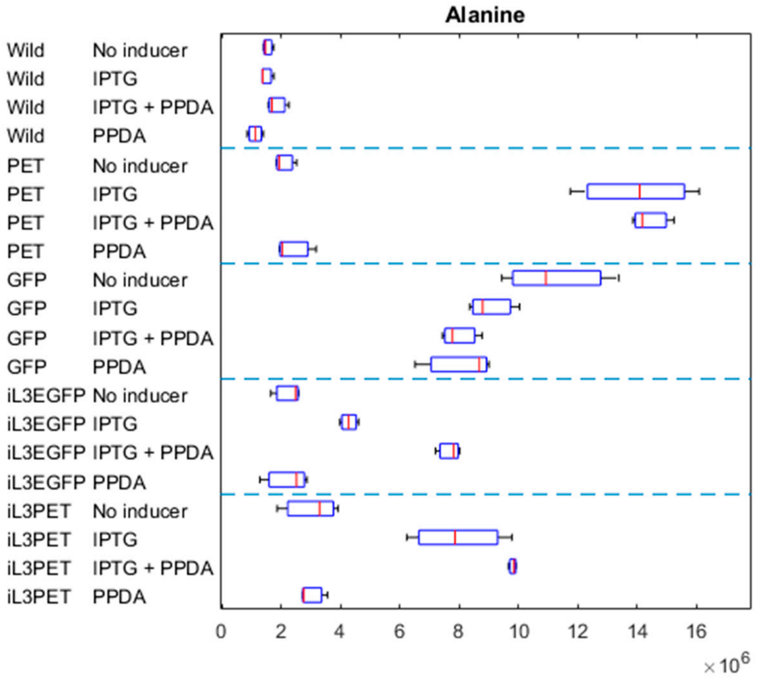

Figure S1. Box-whisker plot of alanine in *riboswitch* data set.

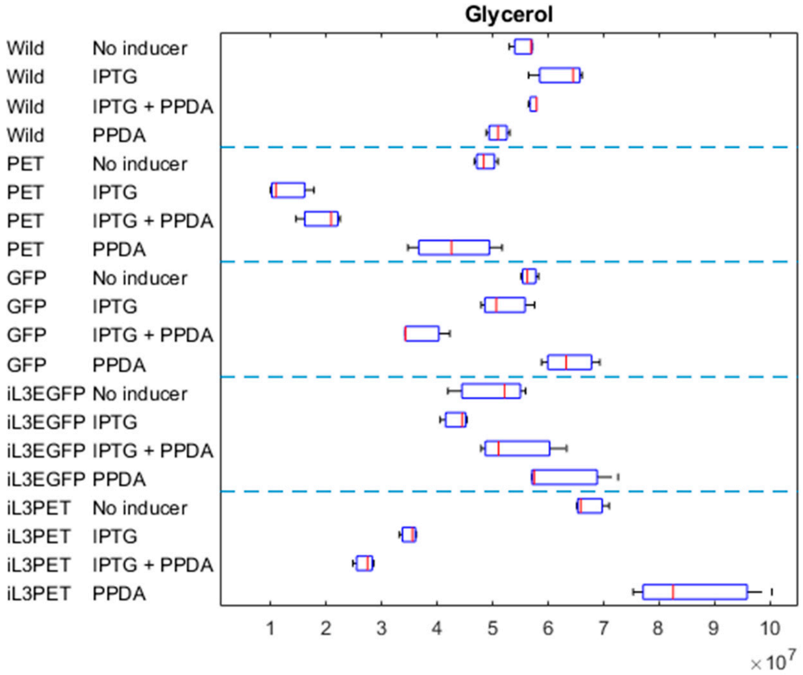

Figure S2. Box-whisker plot of glycerol in *riboswitch* data set.

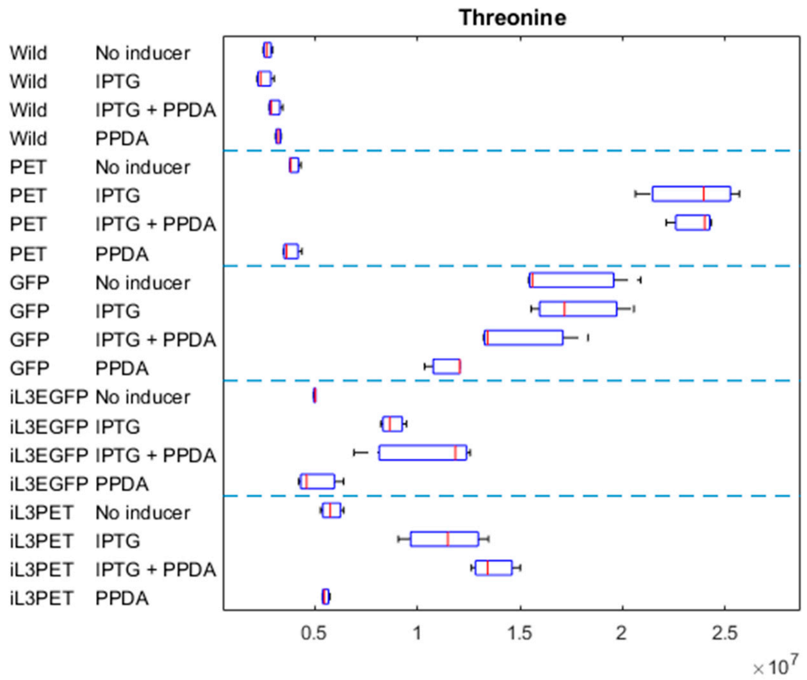

Figure S3. Box-whisker plot of threonine in *riboswitch* data set.

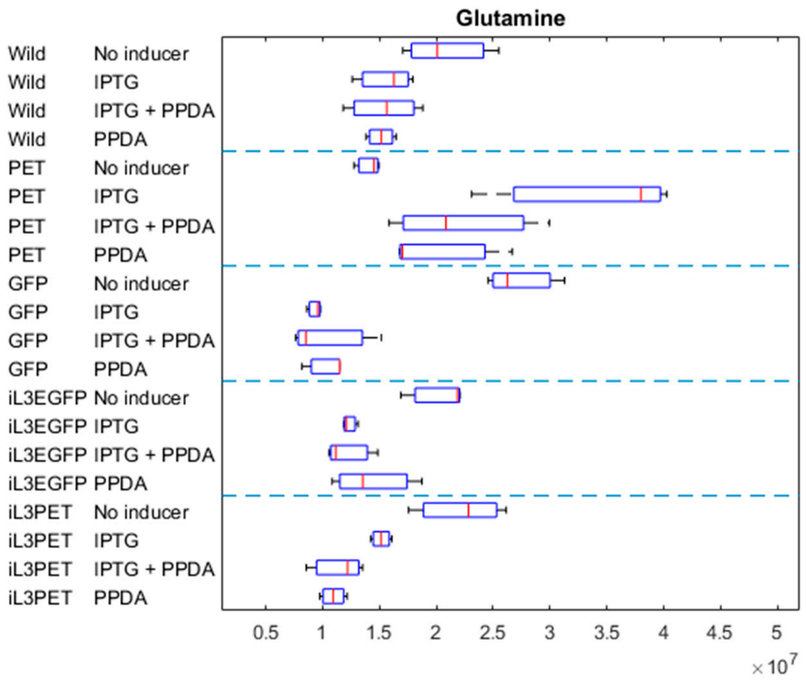

Figure S4. Box-whisker plot of glutamine in *riboswitch* data set.

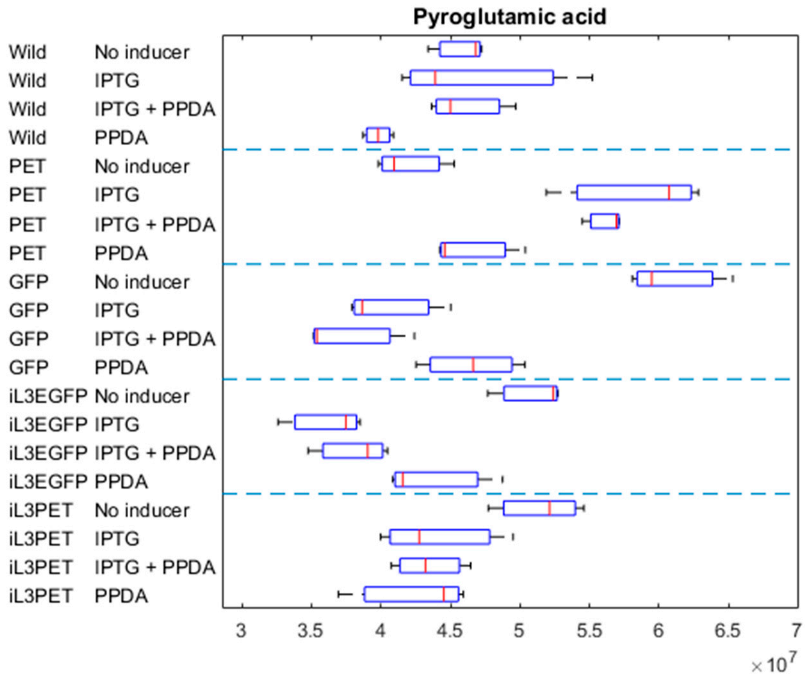

Figure S5. Box-whisker plot of pyroglutamic acid in *riboswitch* data set.

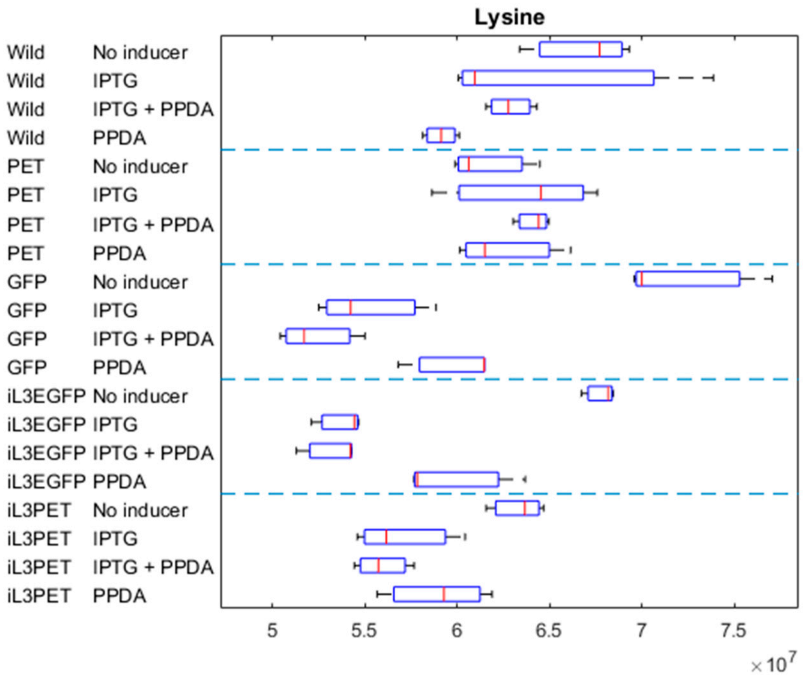

Figure S6. Box-whisker plot of lysine in *riboswitch* data set.

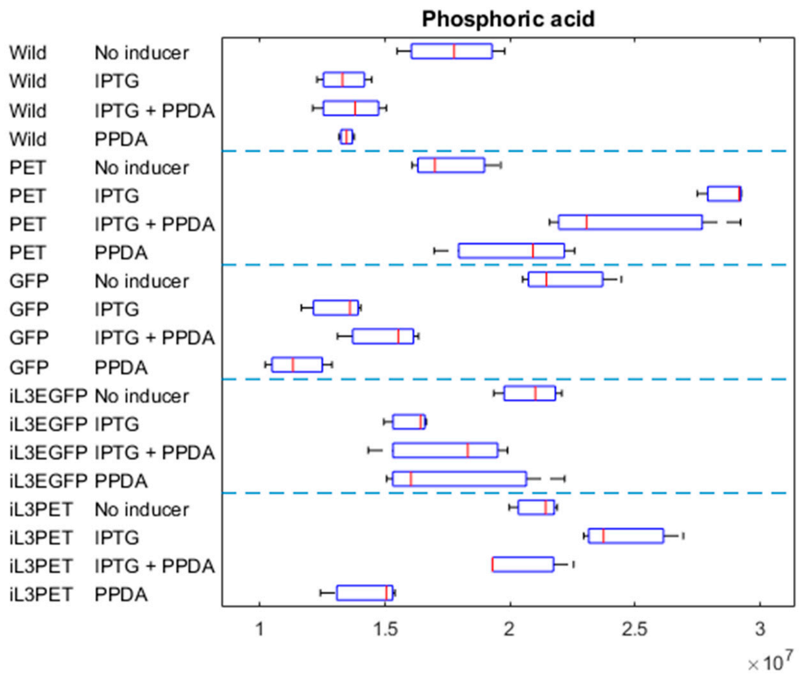

Figure S7. Box-whisker plot of phosphoric acid in *riboswitch* data set.

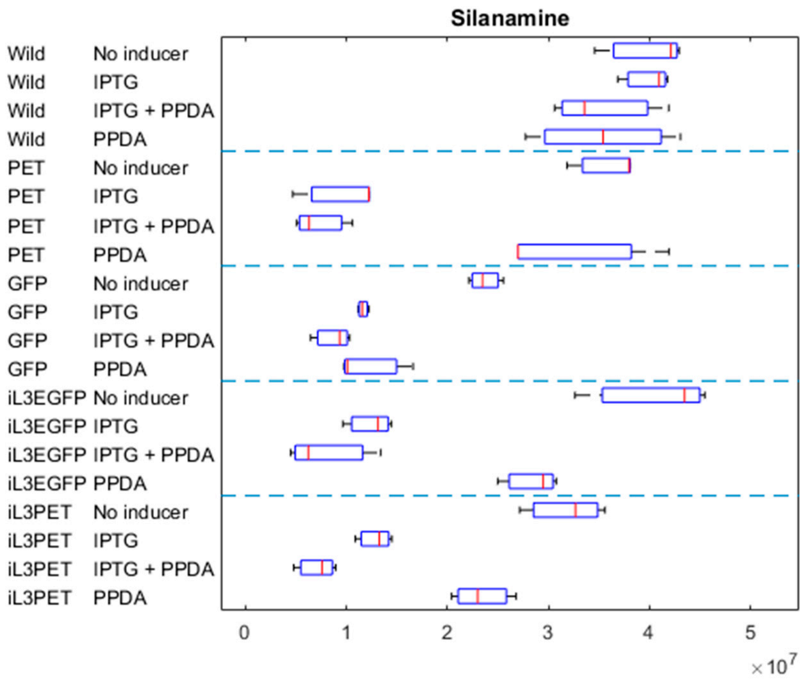

Figure S8. Box-whisker plot of silanamine in *riboswitch* data set.

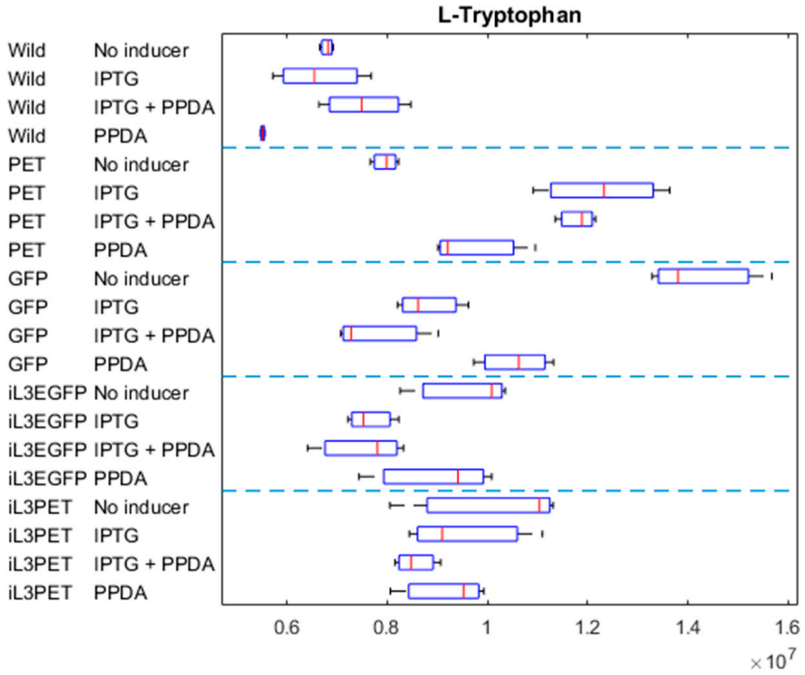

Figure S9. Box-whisker plot of L-tryptophan in *riboswitch* data set.

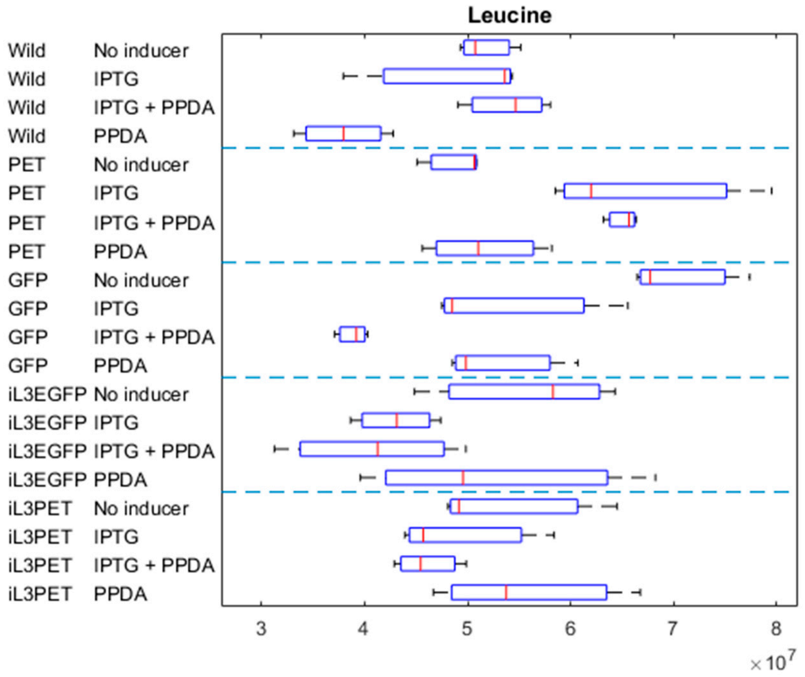

Figure S10. Box-whisker plot of leucine in *riboswitch* data set.

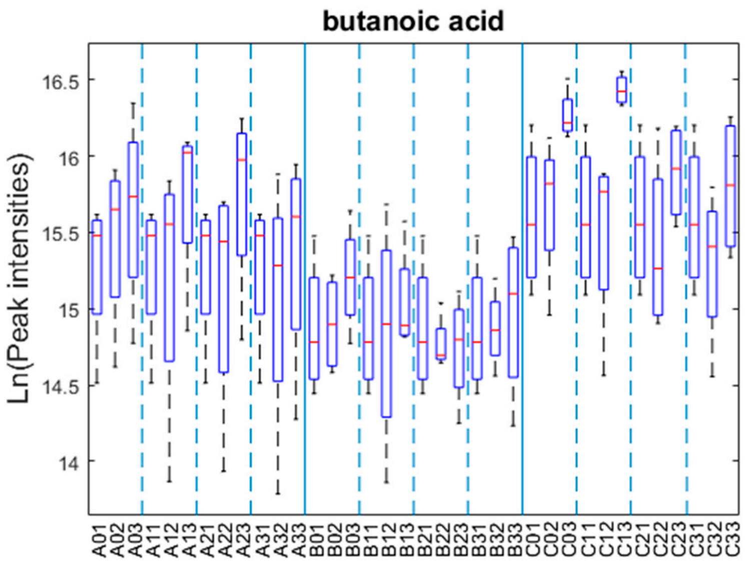

Figure S11. Box-whisker plot of butanoic acid in *propranolol* data set. Dashed lines separate different dosages of propranolol and the solid lines separate different strains. The label is constructed in a format of "Aac", "A" represents strains, varies from A to C: A = *P. putida* DOT-T1E; B = *P. putida* DOT-T1E-PS28; and C = *P. putida* DOT-T1E-18. "a" represents four different concentration levels, and varies from 0–3: 0 = control; 1 = 0.2 mg·mL^{-1}; 2 = 0.4 mg·mL^{-1}; and 3 = 0.6 mg·mL^{-1} propranolol. "c" represents time points, 1 = T0 (0 min); 2 = T1 (10 min) and 3 = T2 (1 h). Such plots give a comprehensive view of how the concentration levels of the metabolite changes under each unique combination of the factors (strains, dosage of propranolol, and time). Note that T0 samples of the same type of bacteria are the same as the bacteria before the exposure to the drug. Therefore A01, A11, A21, and A31 are of the same type; B01, B11, B21, and A31 are the same; and C01, C11, C21 and C31 are the same.

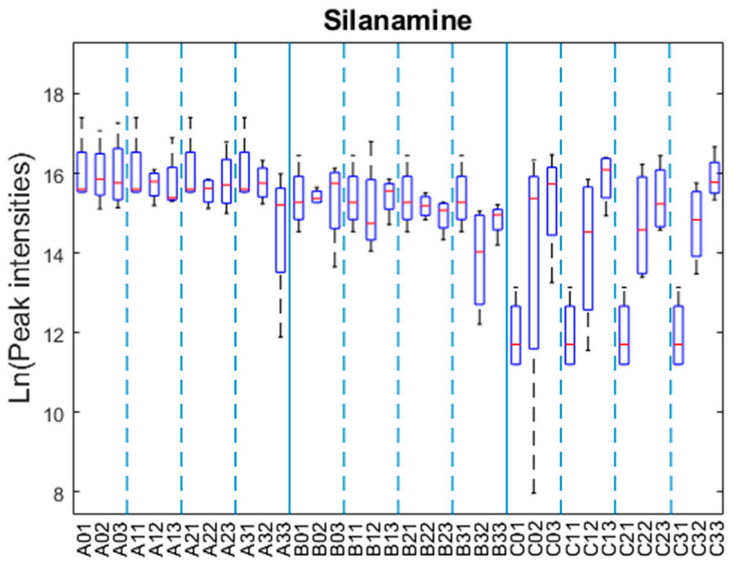

Figure S12. Box-whisker plot of silanamine in *propranolol* data set. Lines and labels presented follow the same rule as described in Figure S11.

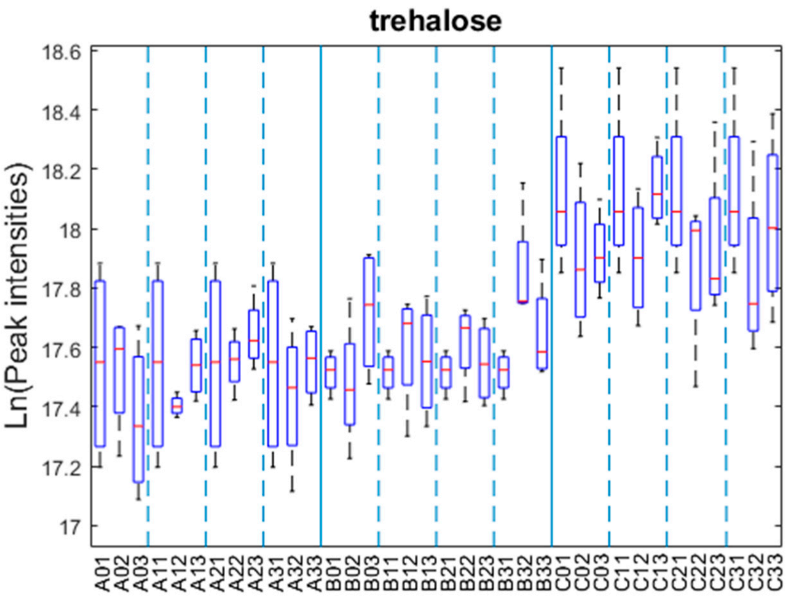

Figure S13. Box-whisker plot of trehalose in *propranolol* data set. Lines and labels presented follow the same rule as described in Figure S11.

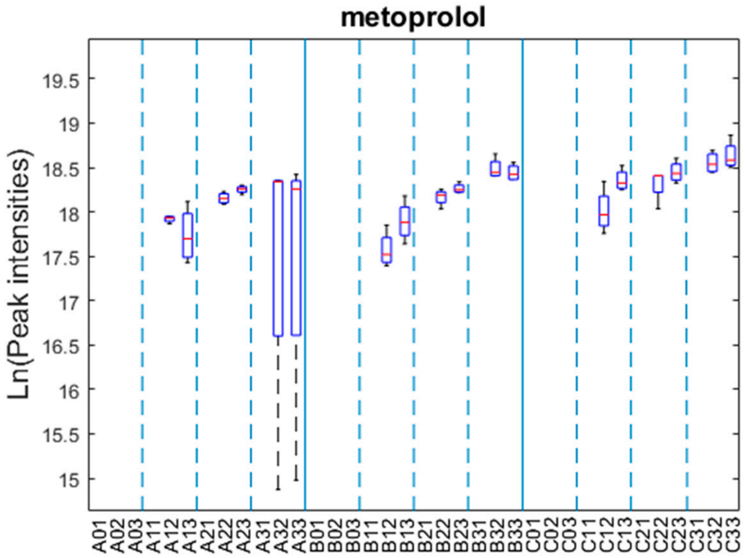

Figure S14. Box-whisker plot of metoprolol in *propranolol* data set. Lines and labels presented follow the same rule as described in Figure S11.

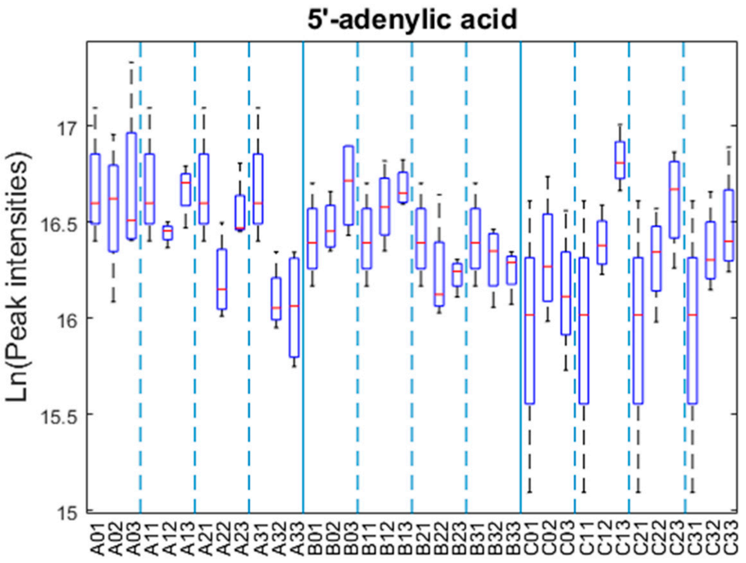

Figure S15. Box-whisker plot of 5′-adenylic acid in *propranolol* data set. Lines and labels presented follow the same rule as described in Figure S11.

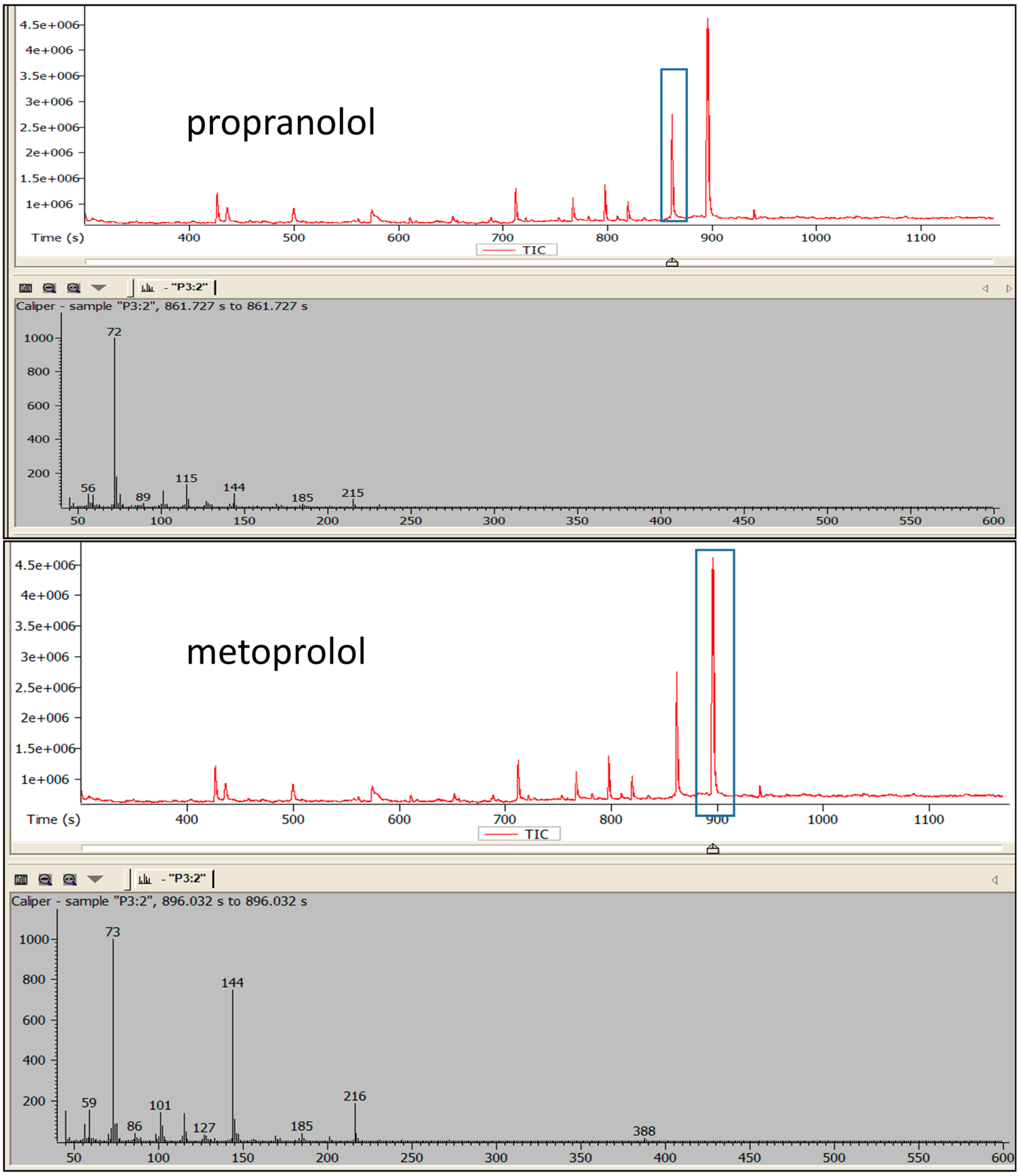

Figure S16. GC-MS analysis on propranolol standards.
